# Improvement and Evaluation of the TOPCOP Taxonomy of Patient Portals: Taxonomy-Evaluation-Delphi (TED) Approach

**DOI:** 10.2196/30701

**Published:** 2021-10-05

**Authors:** Michael Glöggler, Elske Ammenwerth

**Affiliations:** 1 Institute of Medical Informatics UMIT – Private University for Health Sciences, Medical Informatics and Technology Hall in Tirol Austria

**Keywords:** taxonomy, classification system, patient portal, EHR portal, online EHR access, evaluation, Delphi study, electronic health records, digital health, health information, information management, user perspectives

## Abstract

**Background:**

Patient portals have been introduced in many countries over the last 10 years, but many health information managers still feel they have too little knowledge of patient portals. A taxonomy can help them to better compare and select portals. This has led us to develop the TOPCOP taxonomy for classifying and comparing patient portals. However, the taxonomy has not been evaluated by users.

**Objective:**

This study aimed to evaluate the taxonomy’s usefulness to support health information managers in comparing, classifying, defining a requirement profile for, and selecting patient portals and to improve the taxonomy where needed.

**Methods:**

We used a modified Delphi approach. We sampled a heterogeneous panel of 13 health information managers from 3 countries using the criterion sampling strategy. We conducted 4 anonymous survey rounds with qualitative and quantitative questions. In round 1, the panelists assessed the appropriateness of each dimension, and we collected new ideas to improve the dimensions. In rounds 2 and 3, the panelists iteratively evaluated the taxonomy that was revised based on round 1. In round 4, the panelists assessed the need for a taxonomy and the appropriateness of patient engagement as a distinguishing concept. Then, they compared 2 real portals with the final taxonomy and evaluated its usefulness for comparing portals, creating an initial requirement profile, and selecting patient portals. To determine group consensus, we applied the RAND/UCLA Appropriateness Method.

**Results:**

The final taxonomy consists of 25 dimensions with 65 characteristics. Five new dimensions were added to the original taxonomy, with 8 characteristics added to already existing dimensions. Group consensus was achieved on the need for such a taxonomy to compare portals, on patient engagement as an appropriate distinguishing concept, and on the comprehensibility of the taxonomy’s form. Further, consensus was achieved on the taxonomy’s usefulness for classifying and comparing portals, assisting users in better understanding portals, creating a requirement profile, and selecting portals. This allowed us to test the usefulness of the final taxonomy with the intended users.

**Conclusions:**

The TOPCOP taxonomy aims to support health information managers in comparing and selecting patient portals. By providing a standardized terminology to describe various aspects of patient portals independent of clinical setting or country, the taxonomy will also be useful for advancing research and evaluation of patient portals.

## Introduction

### Background

The delivery of knowledge-based care depends on patient engagement, where patients take an active role in their care [1-3]. Patient portals are considered a health information technology that promotes patient engagement [4-6] by providing patients with online tools to take an active role in their care [7-9]. Since patient portals are more than just static repositories for patient data [10,11], they support the new vision of health services that enable patient-provider information sharing [12,13], thus contributing to empowering patients [14,15], supporting shared decision making [16], and engaging patients actively in their care [6,17]. A patient portal is an internet-based application combining knowledge and software tools [18,19] that allow patients to have autonomous access to their electronic health record (EHR) anywhere at any time [20,21]. Besides its core function of providing EHR access [22], the features of a patient portal range from viewing visit notes, requesting medication refills, appointment scheduling, access to test and lab results, secure messaging with the health provider, e-visits, or reporting patient-generated health data [17,23-26]. Patient portals are used in different organizational settings such as independent physician practices and hospitals, group practices, or large, integrated health care delivery organizations [27-29].

### The Need for a Taxonomy of Patient Portals

The widespread use of the internet, rise of mobile computing, and progress in patients’ technical aptness have led to an increase in the use of patient portals in various countries such as the United States, Denmark, and Australia [12,28,30]. However, there are countries where patient portals are still not widely used [31,32]. A benchmarking study presented by Ammenwerth et al [33] in 2020 analyzed the eHealth progress of 14 countries worldwide with different health systems and different levels of economic development. The study showed that the use of patient portals and the provided patient portal functionalities vary significantly between the countries. While Finland and South Korea, for example, allow patients the best access to their health record data, 6 of the 14 analyzed countries do not offer their population any access to their health data online [33].

The low use of patient portals in both developing and high-income countries [31,34] creates a problematic situation for the health informatics professionals who are responsible for strategic and tactical information technology management in their health care institution or department; we will call these professionals “health information managers.” On the one hand, there already exists a very heterogeneous landscape and a broad diversity of patient portals [12,27,35,36] regarding their intended deployment and functionalities [25,30,37]. In contrast, many health information managers still feel they have too little knowledge of patient portals [32]. They admit having difficulties understanding the portals’ various application areas and scopes, defining their general requirements, and selecting a patient portal for their specific context or problem [32]. Health information managers are responsible for planning, organizing, and following up on all activities related to health information technology [38,39]. This also involves selecting, introducing, and managing patient portals for their health care institution [32].

**Figure 1:**
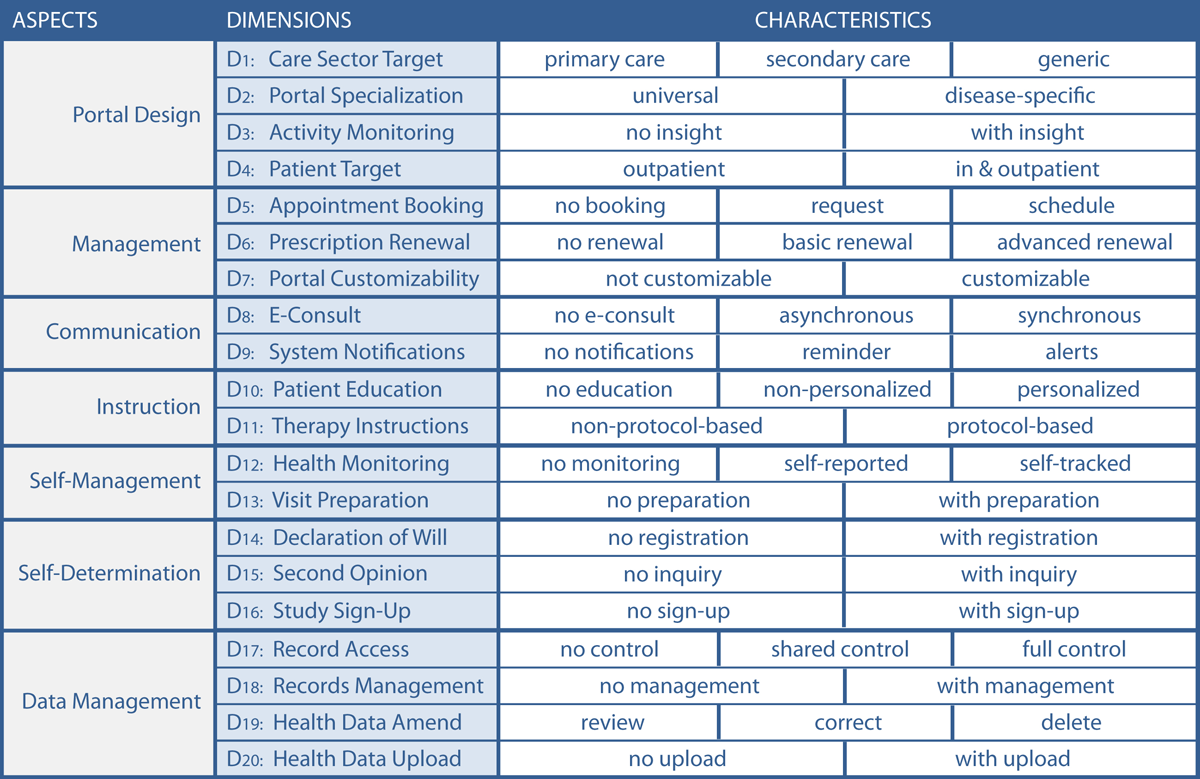
The TOPCOP taxonomy of patient portals [32]: A patient portal is described regarding 7 aspects that cover 20 dimensions. A patient portal can be described by selecting 1 characteristic per dimension.

To support health information managers with a tool for comparing patient portals and defining which general type and functionalities of patient portals they need, we developed the TOPCOP taxonomy (Taxonomy of Patient Portals based on Characteristics of Patient Engagement) [32]. The need for such a patient portal taxonomy had already been stressed in a recently published Cochrane Review on the impact of patient portals [40]. The TOPCOP taxonomy is shown in Figure 1. A comprehensive description of the dimensions is published elsewhere [32].

### The Development of the TOPCOP Taxonomy

A taxonomy is a classification system to classify similar objects of a domain into groups based on distinct characteristics and offers a set of decision rules [41-44]. The reduction of complexity, identifying similarities and differences among objects [45,46], and the understanding of interrelationships are major advantages of taxonomies [44,47,48]. Taxonomies thus support researchers and practitioners in better understanding a domain and distinguishing among its objects [49,50]. The TOPCOP taxonomy was built by applying the formal taxonomy-building method proposed by Nickerson et al [51]. This method specifies the necessary steps and integrates 2 optional, iterative development approaches to conceptionally and empirically build and empirically evaluate a taxonomy [51,52]. The TOPCOP taxonomy was created conceptionally based on a literature review to assess the characteristics and functionalities of patient portals [32]. It was evaluated empirically by classifying patient portals offered on the market and health providers’ portals online [32].

A taxonomy is determined by the user’s intended purpose, which guides the taxonomy’s development by focusing on the specific phenomenon of interest [50,53]. Different users or different purposes may therefore lead to a different taxonomy [51,54]. We created the TOPCOP taxonomy for health information managers to classify and compare patient portals [32]. Further, the taxonomy should serve the health information managers in defining the general type and functionalities of patient portals and should help them select the most suitable solution offered on the market. The dimensions were built to distinguish among patient portals with the scope on patient engagement.

Since there is no objective metric to define the usefulness or quality of a taxonomy [44,51,55], the method by Nickerson et al [51] provides a set of conditions to determine usefulness. Applying these conditions during the building process, the taxonomy’s usefulness was empirically validated by classifying all patient portals of interest with the taxonomy [32].

### The Requirement for a Taxonomy’s Evaluation

According to design science research, taxonomies are fundamental design artifacts to provide knowledge and understanding of a problem domain [49]. In design science research, the design process for an artifact is divided first into building and then into evaluating [56,57]. Following the design science research paradigm, one suitable criterion to evaluate a taxonomy is by having users assess its usefulness in achieving its intended purpose [58,59]. Therefore, we now wanted to evaluate and further improve the TOPCOP taxonomy together with the projected users, guided by the evaluation criteria of the taxonomy’s usefulness related to its intended purpose.

## Methods

### The Delphi Technique

#### Overview

We applied a modified Delphi approach to evaluate the TOPCOP taxonomy. The Delphi technique is a qualitative method, first described by Dalkey and Helmer [60]. It is used in many research areas such as business, policy science, education, health sciences, information science, and health informatics [61-65]. Since there is no consistency in the methods used for evaluating a taxonomy [49], we opted for the Delphi technique because it is commonly agreed that Delphi research elicits sound scientific evidence [66]. Further, various researchers argue that qualitative methods may be particularly appropriate for evaluating design artifacts [67] including taxonomies.

The Delphi technique was particularly suited for our study as it aims to obtain a highly reliable consensus of group opinions on the research items [68] and has been used by researchers to evaluate taxonomies in the past [69-73]. The method is adequate to explore a domain [63], elicit new evidence, and generate new ideas [68,74]. Since the aim of this study was to collect new ideas on dimensions, improve the existing TOPCOP taxonomy, and achieve consensus on the appropriateness of the dimensions by the taxonomy’s users, we considered Delphi to be the best approach as it goes beyond collecting simple intuitive expert opinions [75]. Further, the method applies relatively rigorous control over the interviewing methods, the controlled opinion feedback, and the summary of the results [76].

Using a series of survey rounds delivered in multiple iterations, interspersed with controlled opinion feedback [76], we were able to collect new ideas and correlate the panelists’ opinions on our research items anonymously to improve the taxonomy [60,62,75]. Further, the method is highly flexible [77], accommodating many variations [78-80], and can be used to conduct evaluation studies [81], allowing us to adapt and modify the technique to evaluate our taxonomy. While the classic Delphi aims to generate opinions from experts to make forecasts [60,82], we wanted to collect and correlate the opinions of the taxonomy’s users to improve and evaluate the TOPCOP taxonomy. We now outline the methodological approaches applied to our study.

#### Selection of the Panelists

There are no agreed standards on how to select the participants for Delphi studies [75,83]. We applied a criterion sampling strategy, which is a preferred approach in many Delphi studies [84]. Since the taxonomy should help health information managers to compare patient portals, eligible individuals had to hold a role within a health provider’s organization where they would be actively involved in a patient portal’s selection process. We applied the snowball method [85] to reach out to potential panelists involving patient portal vendors known from the taxonomy development phase [32]. The snowball method has been legitimized and used in many other Delphi studies [75,86,87]. Since there is no standard method for identifying the best number of individuals for inclusion in a Delphi study [86,87], we determined this number based on our research aim and availability of expertise as proposed by several researchers [88-90].

We selected 13 health information managers from Germany, Switzerland, and Austria as we wanted to include panelists from countries with diverse health systems and different progress in eHealth [31,33]. By sampling panelists from different countries but who speak the same language, we aimed to avoid a possible language bias as the survey questions and the complex explanations of the dimensions could otherwise be misinterpreted [91]. Since homogeneous panels tend to find consensus more quickly than heterogeneous panels [92,93], we wanted to enhance credibility through diversity, considering the broadest possible range of participants’ experiences with patient portals and geographic diversity to depict the real situation of different health systems. The educational backgrounds of health information managers are not uniform but may be diverse [39]. We, therefore, included panelists with different educational backgrounds in our sample. In Table 1, we present the final panel.

**Table 1:** Study sample selection.

Panelist number	Country	Gender	AE^a^	PB^b^	Role	WE^c^ (years)	EPP^d^ (years)	INST^e^	SPP^f^
1	Austria	Male	MEng	Computer engineer	Head of department	27	5	HCP^g^	Yes
2	Austria	Male	BEng	Computer scientist	Head of department	15	5	HCP	Yes
3	Austria	Female	BEng	Medical informatics	Project manager	26	1	HCP	Yes
4	Austria	Male	PhD	Electronics engineer	Head of department	25	1	HCP	Yes
5	Austria	Male	MEng	Medical informatics	Head of department	12	1	HCP	Yes
6	Austria	Male	BSc	Bioengineering	Head of department	33	10	HCP	Yes
7	Switzerland	Male	BEng	Medical informatics	System engineer	10	16	HCP	Yes
8	Switzerland	Female	MSc	eHealth management	Researcher	13	3	HCO^h^	No
9	Switzerland	Male	MSc	Medical informatics	Head of department	28	2	HCP	Yes
10	Germany	Male	BSc	System engineer	Head of department	20	7	HCP	Yes
11	Germany	Male	PhD	Medical informatics	Researcher	10	6	HCP	Yes
12	Germany	Male	MSc	Medical informatics	Head of eHealth	13	8	HCO	No
13	Germany	Female	MD	Physician	Head of eHealth	26	8	HCP	Yes

^a^AE: academic education.

^b^PB: professional background.

^c^WE: work experience.

^d^EPP: experience with patient portals.

^e^INST: institution.

^f^SPP: would be involved in selecting a patient portal.

^g^HCP: health care provider.

^h^HCO: health care organization.

#### Determination of the Number of Survey Rounds for Evaluation

As per common agreement, the number of survey rounds is guided by the nature of the study and the level of consensus achieved among the participants during each iteration [75,76,94]. Our study was planned to be performed in 4 assessment cycles, guided by the elicitation of evidence and the achievement of group consensus. In the first 3 rounds, we aimed to collect ideas to improve the taxonomy and to achieve group consensus on every single dimension of the taxonomy related to its appropriateness for comparing patient portals. In round 4, the panelists were asked to evaluate the final taxonomy as a whole as proposed by Wiliam and Black [95], related to its intended use. The survey rounds were performed between January 2021 and April 2021.

#### Achievement of Consensus—the RAND/UCLA Appropriateness Method

The goal of the Delphi technique is to achieve a consensus of opinions from a group of individuals concerning a particular topic or task [87,96,97]. However, there is no general agreement on what statistical aggregation or method is best to determine consensus [98]. Since we wanted to assess the appropriateness of the taxonomy, we considered the concept of the RAND/UCLA Appropriateness Method, called RAM [99], most suitable to determine achievement of consensus in our study. While the RAM method is widely used to determine the appropriateness of health care services [100,101], we applied the model’s consensus measure to evaluate the appropriateness of the taxonomy’s dimensions for classifying and comparing patient portals.

The RAM method uses the median to measure the central tendency of the panelists’ ratings, and ratings should be spread over a 1-9 rating scale [89,99]. The RAM method offers various conditions to constitute disagreement of opinions [99], from which we chose DS9, the strictest definition of disagreement [99]. DS9 means that a dimension is appropriate for comparing patient portals if group consensus with a median of 7-9 without disagreement is achieved. Considering all ratings, disagreement exists when at least one rating is a 1 and at least one is a 9. A dimension is considered uncertain for comparing patient portals if group consensus achieves a median of 4-6 or if there is any median with disagreement. A dimension is considered inappropriate for comparing patient portals if group consensus achieves a median of 1-3 without disagreement. The DS9 measure was applied for all assessments to determine achievement of group consensus. The DS9 measure is summarized in Table 2.

**Table 2:** The DS9 RAND/UCLA Appropriateness Measure with dispersion: considering all ratings, at least one is a 1, and at least one is a 9.

Appropriateness	Panel median	Dispersion condition
Appropriate	7-9	Without disagreement
Uncertain	4-6	Or any median with disagreement
Inappropriate	1-3	Without disagreement

#### Applying Anonymity to Express Opinions Freely

The complete study was conducted anonymously, which means that none of our panelists knew who participated in the survey and no interaction was possible between them. Anonymity allowed greater freedom for our panelists to express their views [102,103] and opinions freely as it avoids the problem of dominant contributors possibly influencing individual opinions [104,105].

#### Introductory Conversations to Enhance Adherence of Panelists and to Create a Common Understanding of the Research Topics

We were aware of the known problem that participants might drop out [77] due to the time-consuming commitment, unforeseen shortage of time, loss of interest, or distraction between the rounds, risking a poor response rate [83]. To promote motivation and strengthen adherence, we conducted an introductory conversation with each panelist separately, as proposed by Daniel and White [106], using the Zoom video conferencing tool [107]. The scope was to give the panelists the possibility to ask questions related to the aim of the study, the research process, and their role in the study and to create a common understanding on all topics.

#### Set-Up of the Online Survey

**Figure 2:**
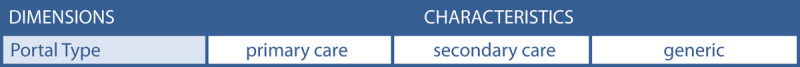
Example for the display of a single dimension for rating.

All interviews were carried out with online questionnaires using a commercial survey product [108]. The survey was piloted by 4 different persons other than the researchers. The survey contained quantitative and qualitative questions. The quantitative ratings served to assess the dimensions’ appropriateness (rounds 1-3) and the final taxonomy as a whole (round 4). To assess the dimensions, we presented only 1 dimension with its characteristics per page (example Figure 2) and added a comprehensive definition of the existing characteristics and the newly proposed characteristics to assure that all panelists had the same understanding of the dimensions.

We provided a Likert scale ranging from 1 to 9 as proposed by RAM [99] for assessment. The open-ended qualitative questions provided in rounds 1-3 allowed the panelists to comment on their ratings if the rating fell into values between 1 and 6. Further, the qualitative questions allowed the panelists to improve the taxonomy by making proposals for new characteristics or dimensions. All proposals had to follow the knowledge-guiding principle of being suitable for promoting patient engagement. The comments and proposals were presented in the subsequent round for the panelists’ reflection. Comments related to ratings, to changes of existing dimensions, or to new characteristics and dimensions were assigned accordingly. The comments were quoted verbatim with no changes made to the original. At the beginning of each survey round, we provided short guidelines on evaluating the research items and presented the results of the previous round. In round 1, we presented the initial TOPCOP taxonomy (Figure 1). In round 2, we presented the ratings from the first round (Multimedia Appendix 1) demonstrating achievement of group consensus. In round 3, we presented the results from the second round demonstrating for which new characteristics and dimensions group consensus was achieved or not achieved (Multimedia Appendix 2). In round 4, we presented the jointly improved, final taxonomy.

### The 4 Rounds of the TOPCOP Taxonomy’s Improvement and Evaluation

#### Round 1: Assessment of the Existing Dimensions and Proposals for Improvement of the Taxonomy

In the first round, the panelists were asked to assess the appropriateness of the existing TOPCOP taxonomy’s dimensions for classifying and comparing patient portals. Further, they were asked to propose unsuitable or missing characteristics related to the existing dimensions and to suggest new dimensions to improve the taxonomy guided by their needs.

#### Round 2: Assessment of the Newly Proposed Characteristics and Dimensions of Round 1

In the second round, the panelists were first asked to assess the proposals of round 1 for new characteristics to refine existing dimensions. Each proposal was presented with all existing and all new characteristics (Multimedia Appendix 3). Related to the proposals of adding new characteristics to the existing dimensions or merge characteristics, we stressed that the panelists should evaluate the appropriateness of the new or merged characteristic of improving the existing dimension to compare patient portals. Then, the panelists were asked to assess the appropriateness of the new dimensions proposed in round 1 for classifying and comparing patient portals.

#### Round 3: Re-Evaluation of Dimensions Where Group Consensus Was Not Achieved in Previous Rounds

**Figure 3:**
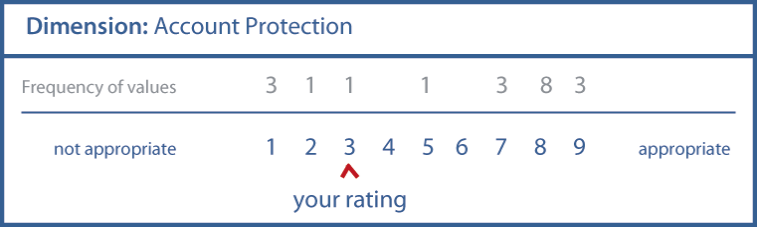
Example of a panelist’s rating in comparison with the group ratings.

In round 3, the panelists were asked to re-evaluate those dimensions and characteristics proposed in round 1 but where no group consensus could be reached in round 2. From some panelists’ comments, we understood that their assessments were guided by national legal requests rather than by evaluating a general area of application. We, therefore, added a note stressing that the scope of this study was to create a generally applicable taxonomy and that specific national requirements should not guide the rating. Since the panelists were to re-evaluate dimensions already assessed in round 2, we provided all panelists with their first rating compared to the group ratings (Figure 3) as recommended by RAM [99]. This was intended to help them better reflect on their rating considering the group opinion.

#### Round 4: Evaluation of the Final TOPCOP Taxonomy as a Whole

Since a taxonomy is complete and adequate when it satisfies the requirements of the purpose for which it was built [58], in round 4, the panelists were asked to evaluate the usefulness of the final taxonomy as a whole [109], related to its intended use. Round 4 was divided into 2 consecutive steps: first performing a case study and then assessing the taxonomy’s usefulness.

First, evaluation is based on comparison [110]. Therefore, the panelists were requested to classify and compare 2 real-world patient portals with the final TOPCOP taxonomy to test its usefulness in a case study. We provided the panelists with 2 anonymized product descriptions from important software companies along with instructions on how to carry out the comparison. Both patient portals could be used for any care sector. However, one was a tethered patient portal while the other was an integrated patient portal. We selected these 2 patient portals because they differ in many characteristics, allowing the panelists to see the taxonomy’s usefulness in comparing very different patient portals.

Second, after the case study was performed, the panelists were asked to assess the usefulness of the taxonomy as a whole. To investigate the panelists’ opinions related to taxonomies for patient portals, we started with the following questions: (Q1) How important do you consider the need for a taxonomy for comparing patient portals? (Q2) How suitable do you consider patient engagement as a guiding concept for comparing patient portals?

Further, the health information managers were asked to make proposals for other guiding concepts that they considered useful to compare patient portals. Since we determined patient engagement as a guiding concept to distinguish among patient portals for the TOPCOP taxonomy, we aimed to collect alternative proposals suitable for future research: (Q3) What other guiding concepts may be appropriate for comparing patient portals?

To assess the taxonomy’s usefulness as a whole [111], 6 research questions related to the performed case study were presented. Since understanding an artifact is a fundamental requirement for its usefulness, the panelists were first asked to evaluate whether the final taxonomy was understandable: (Q4) How understandable is the form and structure of the final taxonomy? Then, they were asked to assess whether the improved TOPCOP taxonomy is useful related to its intended use: (Q5) How useful is the final taxonomy for classifying patient portals following patient engagement? (Q6) How useful is the final taxonomy for comparing patient portals following patient engagement? (Q7) How useful is the final taxonomy for assisting you in better understanding patient portals based on characteristics supporting patient engagement? (Q8) How useful is the final taxonomy for creating an initial requirement profile for patient portals based on characteristics supporting patient engagement? (Q9) How useful is the final taxonomy for selecting patient portals offered on the market based on characteristics supporting patient engagement?

Question Q3 was set up as an open-ended question to collect the panelists’ proposals in the best possible way [112]. To categorize the proposals, we analyzed the responses by applying the summarizing content analysis [113], an inductive analysis method proposed by Mayring [114]. All other items were assessed by applying the RAM approach [99].

## Results

### Results of Round 1: Assessment of the Existing Dimensions and Proposals for Improvement of the Taxonomy

**Figure 4:**
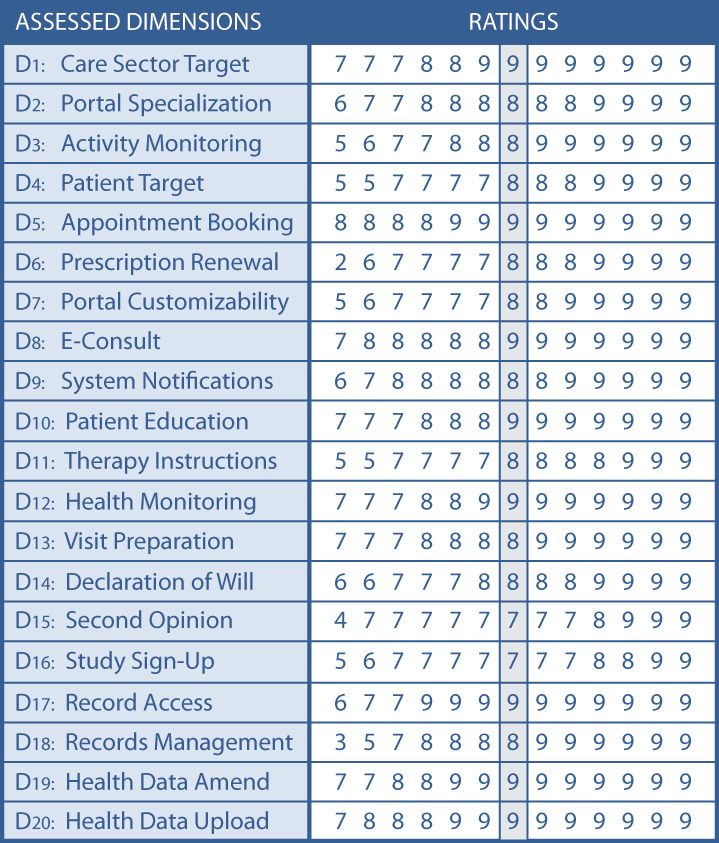
Achieved consensus for the existing dimensions of the TOPCOP taxonomy after round 1. The grey column shows the median value without disagreement. All 13 panelists assessed all dimensions.

The panelists were asked to evaluate the TOPCOP taxonomy by assessing every single dimension related to its appropriateness for classifying and comparing patient portals. In Figure 4, we present the assessment for the 20 dimensions of the initial TOPCOP taxonomy (Figure 1) indicating the median for each dimension. All 13 panelists evaluated all 20 dimensions. Since each dimension’s median ranged between 7 and 9 without disagreement, group consensus on the dimensions’ appropriateness was achieved for all 20 dimensions [99].

Further, the panelists were asked to propose unsuitable and missing characteristics to refine the existing dimensions and to suggest new dimensions to improve the taxonomy.

**Figure 5:**
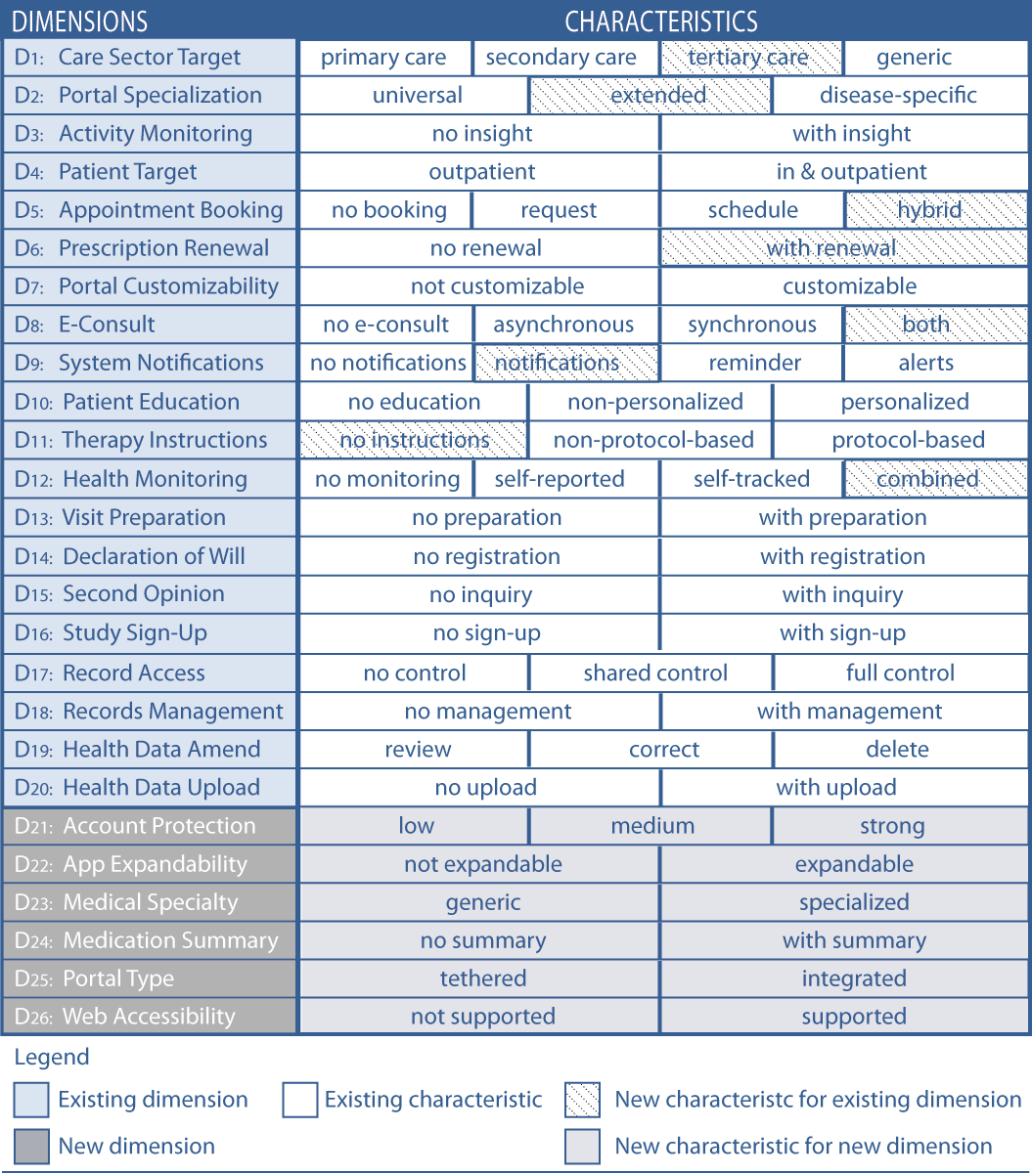
Proposals from round 1 for new characteristics and dimensions to improve the taxonomy.

Two panelists proposed refining dimension D6 Prescription Renewal by merging the characteristics “basic renewal” and “advanced renewal” to create the characteristic “with renewal” instead. They argued that differentiating the dimension into the initial 2 characteristics is confusing rather than strengthening the distinguishability of patient portals. Seven panelists proposed 8 new characteristics to improve the existing dimensions D1, D2, D5, D6, D8, D9, D11, and D12 (Figure 5 shows the content of each dimension). Five panelists proposed the new dimensions Account Protection, App Expandability, Medical Specialty, Medication Summary, Portal Type, and Web Accessibility to enhance the taxonomy. We present all the proposals for improvement of round 1 in Figure 5.

### Results of Round 2: Assessment of the Newly Proposed Characteristics and Dimensions of Round 1

In round 2, the panelists had to assess the proposals from round 1 (Figure 5). They were asked to evaluate the merger of the characteristics of dimension D6. Further, they assessed the appropriateness of the suggested characteristics of dimensions D1, D2, D5, D8, D9, D11, and D12.

**Figure 6:**
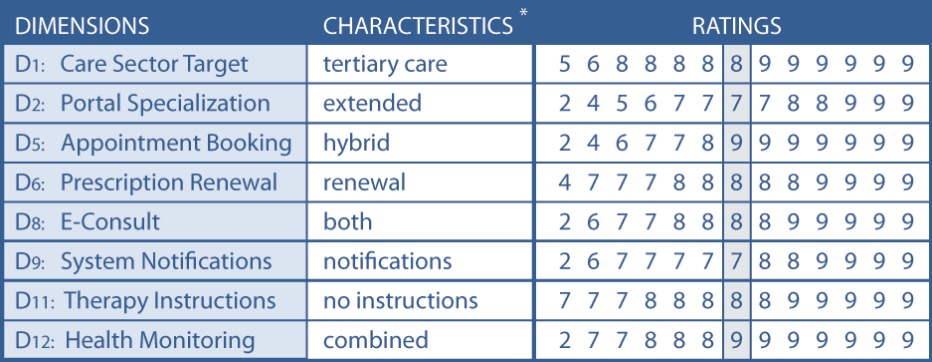
Achieved consensus by all 13 panelists on new characteristics for existing dimensions proposed in round 1. The grey column shows the median without disagreement. *New characteristics to improve the dimension.

All 13 panelists assessed the proposed 8 characteristics and justified their rating whenever it fell between 1 and 6. As demonstrated in Figure 6, the median assessment for the appropriateness of all changes ranged between 7 and 9 without disagreement. Therefore, all 8 characteristics were appropriate for improving the taxonomy and became part of the taxonomy [99].

The panelists were further requested to evaluate the appropriateness of the proposed dimensions D21 Account Protection, D22 App Expandability, D23 Medical Specialty, D24 Medication Summary, D25 Portal Type, and D26 Web Accessibility.

**Figure 7:**
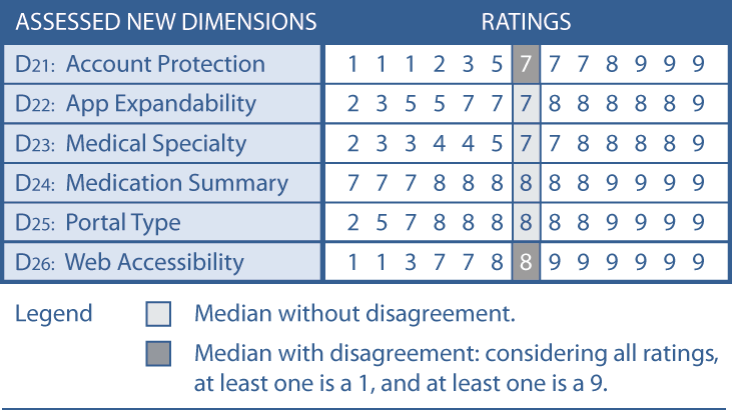
Achieved consensus by all 13 panelists on new dimensions proposed in round 1.

In Figure 7, we demonstrate that for all these dimensions, the median ranged between 7 and 8. However, the condition for disagreement [99] was fulfilled for dimensions D21 Account Protection and D26 Web Accessibility. Therefore, only dimensions D22, D23, D24, and D25 were considered appropriate for improving the taxonomy and became part of the taxonomy. Since no panelist made any proposal for changing an existing dimension or for a new dimension in round 2, only dimensions D21 and D26 became subject to re-evaluation in round 3.

**Figure 8:**
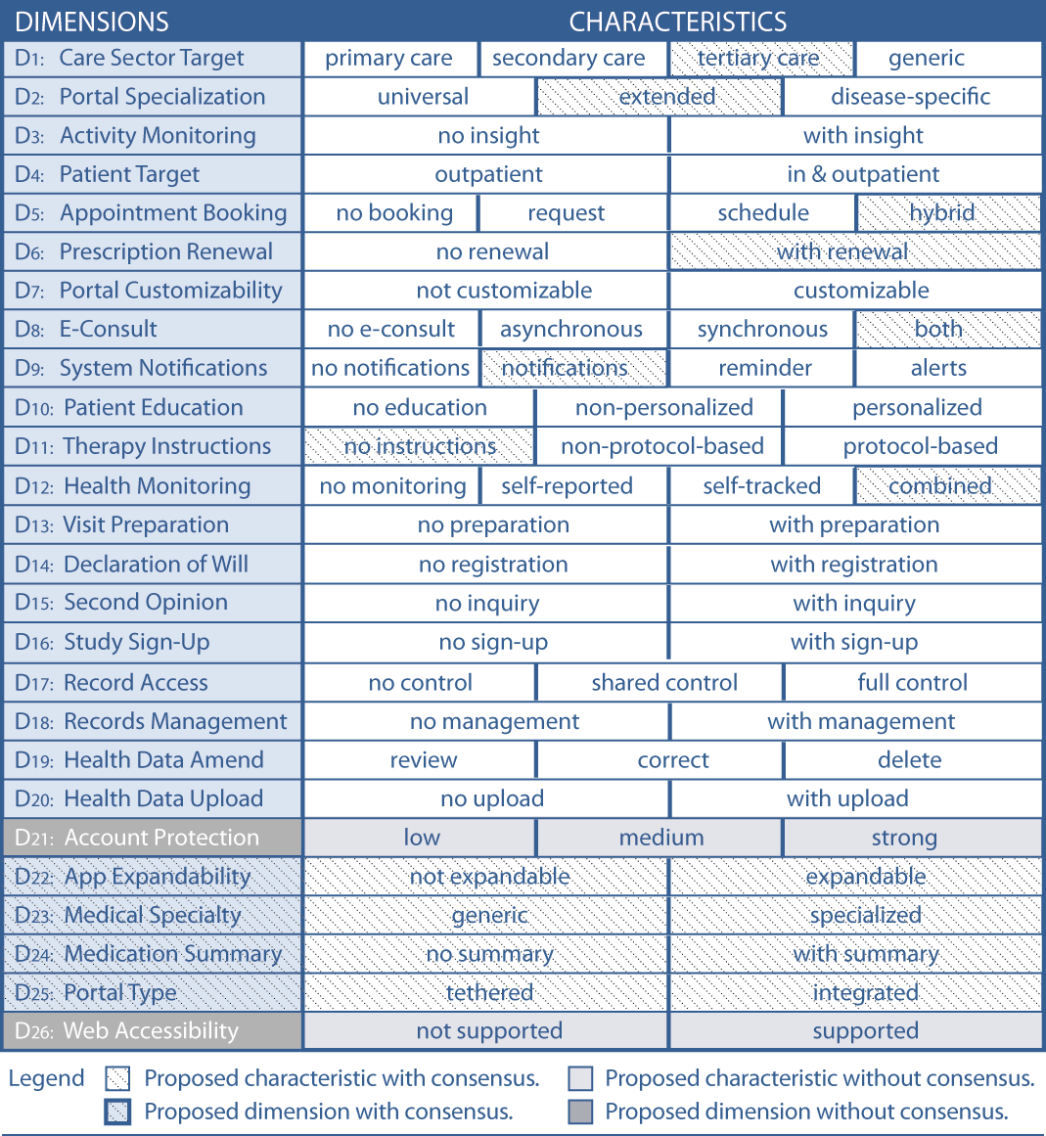
The TOPCOP taxonomy in progress after round 2.

In Figure 8, we present the taxonomy in progress after round 2 showing for which characteristics group consensus was achieved and for which dimensions no group consensus was achieved.

### Results of Round 3: Re-Evaluation of Dimensions Where Group Consensus Was Not Achieved in Previous Rounds

In round 3, the panelists were asked to again assess the new dimensions D21 Account Protection and D26 Web Accessibility as group consensus was not achieved in round 2. All 13 panelists assessed both dimensions.

**Figure 9:**
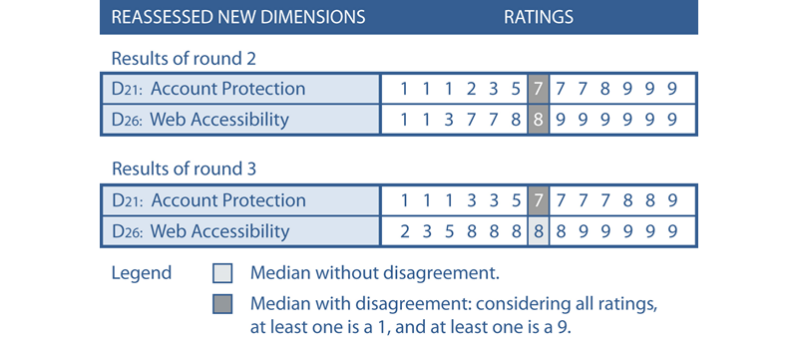
Achieved consensus by all 13 panelists on dimensions D21 and D26 after round 3 in comparison with the consensus in round 2.

As demonstrated in Figure 9, a median of 8 without disagreement [99] was achieved for dimension D26. Dimension D26 was therefore appropriate and became part of the taxonomy. For dimension D21, a median of 7 was achieved. However, as at least one rating is a 1 and at least one rating is a 9, disagreement existed among the panelists [99]. All 5 panelists who assessed dimension D21 with values of 1 and 3 argued consistently that a patient portal must provide the highest data protection due to legal or patient requirements. Therefore, as strong account protection is a mandatory requirement, dimension D21 is not appropriate for distinguishing among patient portals. Comparing the ratings of dimension D21 for rounds 2 and 3 showed that, besides the fact that disagreement was re-confirmed in round 3, 3 ratings deteriorated (Figure 9), which means that group consensus converged even more strongly towards disapproval of dimension D21. To avoid the known risk of fatiguing the panelists with too many evaluation rounds [92], we did not launch another evaluation round. Since no group consensus was achieved, dimension D21 was not integrated into the taxonomy.

**Figure 10:**
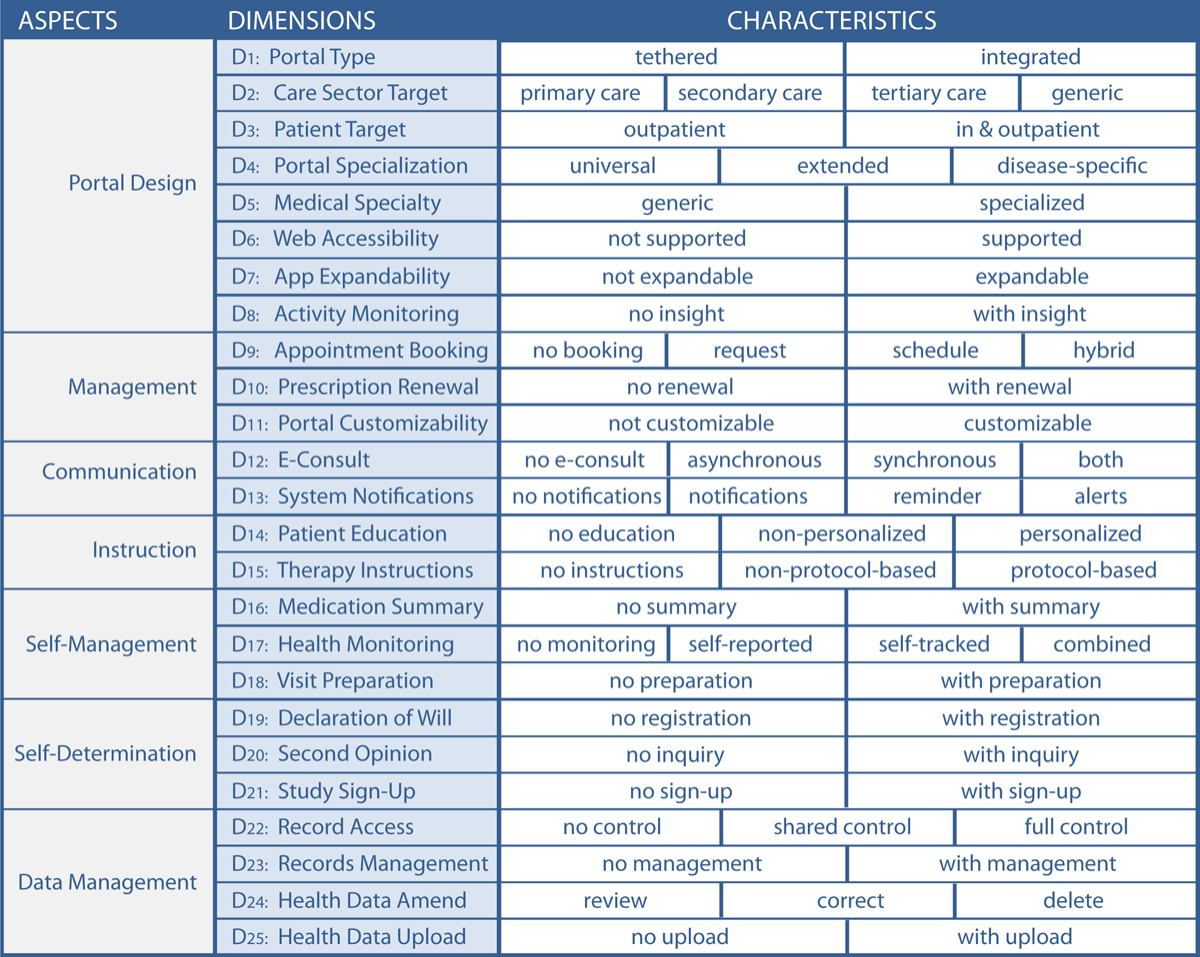
The final and user-evaluated TOPCOP taxonomy of patient portals.

After assigning the new dimensions to suitable aspects and organizing and numbering the dimensions accordingly, the final TOPCOP taxonomy resulted in 25 dimensions based on 65 characteristics assigned to 7 aspects and is presented in Figure 10. In Multimedia Appendix 4, we provide a detailed description of the dimensions and characteristics.

### Results of Round 4: Evaluation of the Final TOPCOP Taxonomy as a Whole

In round 4, the panelists were asked to assess the general need for a taxonomy, the appropriateness of patient engagement as a guiding concept, and the TOPCOP taxonomy’s usefulness related to its intended purpose. All 13 panelists participated in round 4.

**Figure 11:**
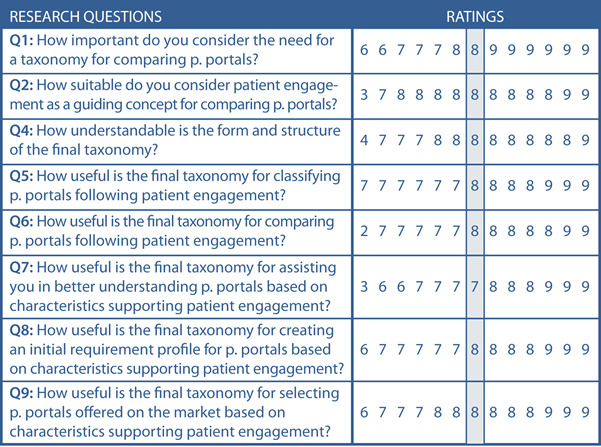
Achieved consensus by all 13 panelists on the research questions Q1, Q2, Q4–Q9. The grey column shows the median without disagreement. p: patient.

In Figure 11, we present the evaluations’ results for the research questions Q1, Q2, and Q4–Q9. Since group consensus was achieved without disagreement [99] for all research questions, no further interview round was launched.

The results shown in Figure 11 can be interpreted as follows. The panelists clearly agreed that there is a need for a taxonomy to distinguish among patient portals (median of 8 for Q1). Twelve panelists considered patient engagement to be an appropriate distinguishing concept for comparing patient portals (median of 8 for Q2). Only the panelist who assessed question Q2 with a rating of 3 proposed “System Architecture, Data Types, and Interoperability” as a more appropriate concept for comparing patient portals. Further, the panelists were asked to propose alternative distinguishing concepts appropriate for comparing patient portals (Q3). Since research question Q3 was an open-ended question, it is not part of Figure 11. Therefore, we present the proposed alternative concepts in Table 3.

**Table 3:** Alternative distinguishing concepts proposed in round 4.

Proposed alternative distinguishing concepts (Q3)	Number of panelists proposing an alternative distinguishing concept
Comparison of patient portals based on characteristics promoting “Health Literacy”	1
Comparison of patient portals based on characteristics supporting “Improvement of Health Outcomes”	1
Comparison of patient portals based on characteristics related to “System Architecture, Data Types, and Interoperability”	3
Comparison of patient portals based on characteristics related to “Improvement of Work Efficiency and Cost Savings”	2

We continued interpreting the results presented in Figure 11, which relate to the case study and the assessment of the taxonomy’s usefulness. With a median of 8, the panelists considered the form and structure of the final taxonomy to be understandable (Q4). However, 1 panelist who assessed Q4 with a rating of 4 argued that the taxonomy contains too many dimensions while, on the contrary, 1 panelist who assessed Q4 with a rating of 7 proposed refining the taxonomy with additional subcharacteristics to achieve a more accurate comparison of patient portals. All panelists considered the final taxonomy to be appropriate for classifying patient portals, giving ratings between 7 and 9 with a median of 8 (Q5). Further, they considered, with a median of 8, the taxonomy to be appropriate for comparing patient portals (Q6) and appropriate for better understanding of patient portals based on characteristics supporting patient engagement (median 7 for Q7).

**Figure 12:**
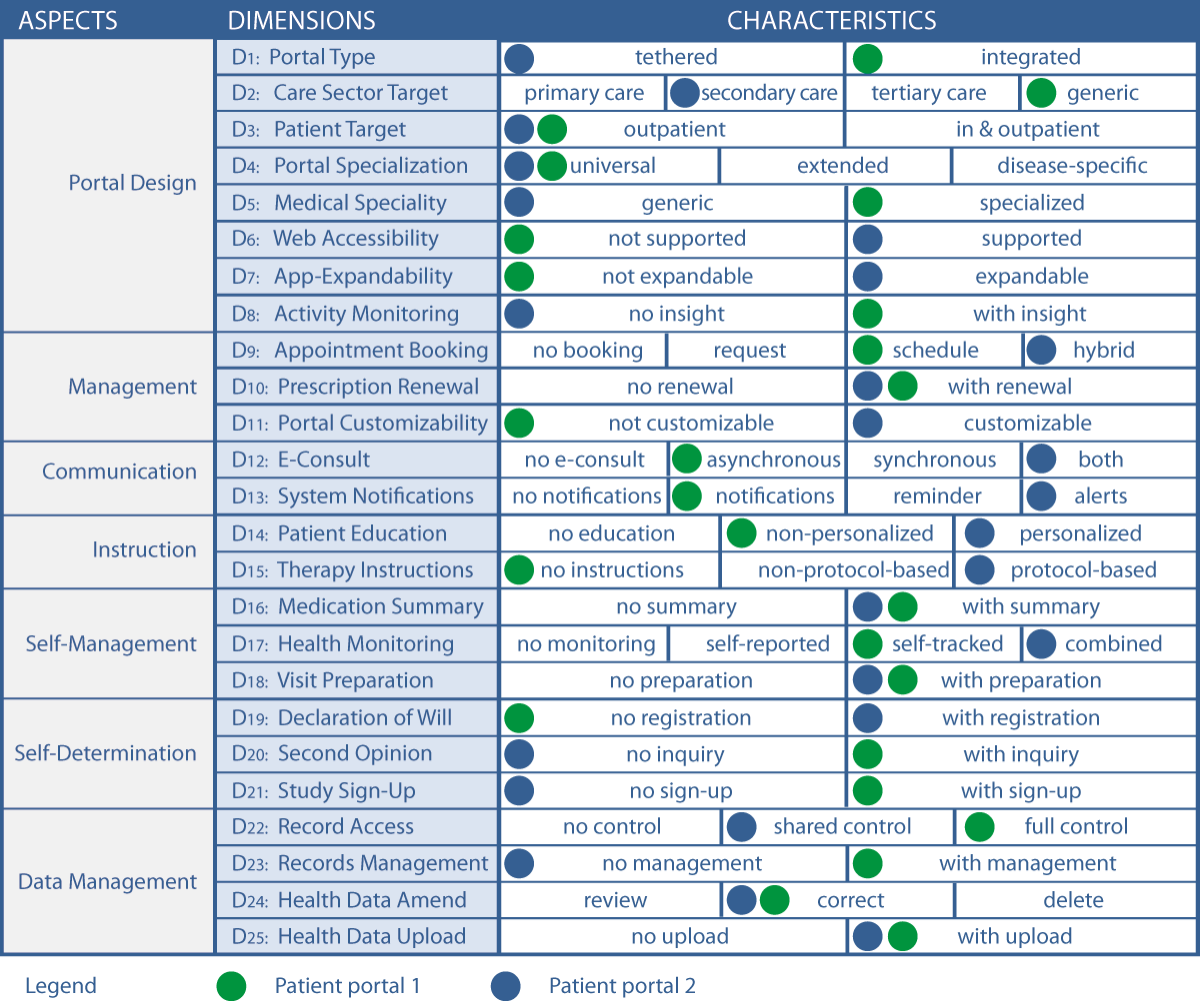
Example of a comparison of 2 real patient portals carried out with the TOPCOP taxonomy by marking the respective characteristics.

Since the TOPCOP taxonomy is also intended to help health information managers select patient portals offered on the market, the panelists were requested to assess its usefulness in this regard. With a median of 8, the group consensus was achieved on both the taxonomy’s usefulness for creating an initial requirement profile for patient portals (Q8) and selecting patient portals offered on the market based on characteristics supporting patient engagement (Q9). To sum up, by applying the TOPCOP taxonomy to compare 2 patient portals, we could indeed show that it is useful in contrasting and comparing patient portals from different vendors. In Figure 12, we show an example of a panelist’s comparison. By marking each patient portal’s characteristics, the differences and similarities of the 2 patient portals could be easily recognized.

### Additional Findings: The Taxonomy-Evaluation-Delphi Approach (TED)

Evaluation is a challenging, essential, and crucial component of the research process [110,115]. One criterion for assessing artifacts such as taxonomies is by evaluating their usefulness related to their intended purpose [111,116]. However, there are only very few taxonomy-specific evaluation guidelines [117], but multiple evaluation approaches can be applied in health informatics [118-120]. Szopinski et al [49] analyzed the various approaches researchers applied to evaluate taxonomies in the information system’s domain and demonstrated that the Delphi technique was hardly used. Analyzing 61 evaluation approaches, they found just 1 study where the Delphi technique was used to evaluate the taxonomy [49]. In this study, panelists were asked to classify items into a deductively developed taxonomy and assess if the items were classified correctly [121]. In contrast, our modified Delphi approach aims to (1) first improve a conceptually and empirically created taxonomy [32] in multiple rounds by the users and (2) then evaluate the jointly created taxonomy by achieving users’ consensus on the usefulness of the taxonomy. Therefore, the health information managers first compared the real-world patient portals with each other by performing a use case and then assessed the taxonomy related to its intended use. In Table 4, we describe the differences between the classic Delphi technique and our approach. The modified Delphi approach we used is, to the best of our knowledge, a new Delphi approach in health informatics for evaluating a taxonomy. We, therefore, call this approach the Taxonomy-Evaluation-Delphi (TED) approach.

**Table 4:** Comparison of the classic Delphi technique with the Taxonomy-Evaluation-Delphi (TED) approach.

Criteria/Delphi	Classic Delphi	TED approach
Objective	To make forecasts to plan ahead [60,82]	To collect new ideas to improve the taxonomy and to have the usefulness of a conceptually and empirically created taxonomy evaluated by the intended users
Approach	Obtain the most reliable consensus on the estimation of numerical quantity [105]	Obtain the most reliable consensus on the taxonomy’s usefulness related to its intended purpose guided by the user’s needs
Anonymity	No strict anonymity [60]	Strict anonymity
Consensus metric	Median without dispersion	Median with dispersion based on the RAM^a^ method [99]
Panelists	Experts^b^ with a deep understanding of the issues of concern [122]	The taxonomy’s users with different levels of experience and understanding of the issue of concern
Number of rounds	Guided by the level of group consensus achieved [75,76,94]	Guided by elicitation of new evidence [95] and the level of group consensus achieved [75,76,94]
Procedure	Questionnaires and follow-up interviews [60]	Introductory conversations, online questionnaires, and performing a case study
Outset	Qualitative questions to collect initial knowledge to create/refine the research subject [60]	Quantitative questions to assess dimensions and characteristics, qualitative questions to collect new ideas to improve the taxonomy, and a case study to compare real patient portals as a basis for the taxonomy’s evaluation
Result	Agreement on numerical quantities [60]	Improved and evaluated useful taxonomy based on the users’ needs

^a^RAM: RAND/UCLA Appropriateness Method.

^b^There is ambiguity regarding the term expert concerning the Delphi technique as there is no unequivocal definition [82,96,123].

## Discussion

### Principal Findings

With this study, we were able to demonstrate that the need for a taxonomy to compare and classify patient portals exists among health information managers and that the concept of patient engagement to compare and select patient portals is considered appropriate.

Applying a modified Delphi approach, we improved the TOPCOP taxonomy based on the specific needs of the users. The final TOPCOP taxonomy consists of 25 dimensions with 65 characteristics, compared to 20 dimensions and 49 characteristics of the initial TOPCOP taxonomy.

We were able to demonstrate that the health information managers considered the final taxonomy to be useful in classifying and comparing patient portals. Further, we demonstrated that the final TOPCOP taxonomy supports the users in better understanding patient portals and assists them in selecting patient portals offered on the market. We were able to collect 4 alternative ideas on distinguishing concepts to compare patient portals that may serve for future research. As an additional outcome of our study, we created, to the best of our knowledge, a new Delphi approach in health informatics for evaluating a taxonomy.

### Comparison With Prior Work

At present, there exists only a limited number of publications related to patient portal taxonomies. Ammenwerth et al [20] developed a taxonomy that aims to distinguish patient portals in a systematic review dealing with their effect on patient empowerment and health-related outcomes. Roehrs et al [124] developed a taxonomy that aims to identify open questions related to personal health record (PHR) data types, features, and architecture types. A PHR provides patients with web-based access to their health data that is under the control of the patient [124], while an EHR typically is under the control of the provider [7,19,22]. Fernández-Alemán et al [125] analyzed free web-based PHRs to identify their features and functions to better understand the PHR market. They created a framework of 4 dimensions intended to support patients in selecting a PHR that best fits their needs [125]. Scheplitz et al [126] created a framework for patient portal functionalities to record all possible functions to identify specification gaps related to software development. Walker et al [8] developed a framework to evaluate how well health information technology can support patient engagement by applying 5 engagement scoring levels.

These attempts only provide part of a potential patient portal taxonomy and are developed for different users and purposes. Since the user determines the intended purpose of a taxonomy and the purpose guides the development by focusing on a specific phenomenon of interest, different users or purposes may lead to different taxonomies [46,51,55]. Further, a useful taxonomy must yield utility for a specific problem domain [58]. To sum up, the found taxonomies are not suitable to yield utility for health information managers for classifying and comparing patient portals based on characteristics appropriate for promoting patient engagement and understanding the differences and similarities. Therefore, the TOPCOP taxonomy was specifically developed for health information managers to compare and select patient portals offered on the market.

### Limitations

Our approach to evaluating the TOPCOP taxonomy has some potential limitations.

First, panelists were selected from Germany, Austria, and Switzerland while the scope of the TOPCOP taxonomy is to support health information managers from any country. Since we were not able to attract participants from other countries, we aimed to assemble the panel as heterogeneously as possible, with different educational backgrounds and work experience, to achieve the best possible understanding of different viewpoints. Further, by selecting participants from 3 countries, we were able to map expertise from 3 different health care systems and integrate experiences with different levels of health care systems’ digitization.

The second limitation relates to the panelists’ experience with patient portals. Patient portals are not widely used in Germany, Austria, or Switzerland [32], and the experience with patient portals varied from little experience to much experience among the selected health information managers. Including participants with little experience with patient portals may lead to different results than if the participants had a deep understanding. As the taxonomy is not intended to only serve highly experienced but also inexperienced users, a composition of the panel that considers different levels of experience and understanding may increase the variety of viewpoints and the range of user needs related to the taxonomy. This variety may make the taxonomy even more useful [127].

The third limitation relates to the risk that the panelists may misunderstand what to evaluate. During the initial phone calls with potential participants, we noticed that some users assumed that the taxonomy’s evaluation related to the suitability of functionalities for patient portals. However, the evaluation related to a dimension’s appropriateness for classifying and comparing patient portals based on patient engagement. To ensure that there was no confusion, we explained the difference in individual introductory video conferences. Further, in the survey’s introductory part, we outlined the scope of the evaluation and formulated the questionnaire’s questions with unambiguous wording.

The fourth limitation is related to the Delphi technique itself. Delphi aims to obtain group consensus on opinions [68], but the achievement of consensus does not necessarily mean that the correct answer was found [84]. Besides, the composition of the panel may influence the research outcome [88,128]. To address these problems, we assembled the panel as heterogeneously as possible to integrate the broadest possible viewpoints and experience with patient portals. The selection of the panel was guided by the goal of achieving the best expertise available.

To determine the achievement of group consensus, we applied the RAND/UCLA concept appropriate for evaluation [101] and widely used to assess the appropriateness of health care services [100,129]. The survey was conducted anonymously to avoid the problem of dominant panelists possibly influencing individual opinions [102]. By applying all these measures, we believe that we were able to reduce any inherent bias in a possible method in the best way.

### Practical Implication

The scope of the TOPCOP taxonomy is to serve health information managers with different degrees of knowledge related to patient portals and for various areas of application. The taxonomy may thus serve health information managers as a starting point to better understand the complex domain of patient portals since it describes the various aspects of patient portals. Further, 2 or more patient portals can be described by marking the respective characteristics. This shows the differences and similarities of the patient portals (Figure 12) and so supports the health information managers in classifying and comparing patient portals.

Since each health care institution may have different requirements related to a patient portal, the TOPCOP taxonomy can serve to create a requirement profile. By marking those characteristics in the taxonomy that best meet the needs of a health care institution, health information managers can create an initial requirement profile. This profile can then be used for a targeted search and selection of suitable portals offered on the market.

By providing a standardized terminology to describe various aspects of patient portals independent of clinical setting or country, the TOPCOP taxonomy is also useful for advancing research and evaluation of patient portals. It can, for example, be used to systematically describe patient portals as part of systematic reviews on their impact. The need for a taxonomy in this context has already been stressed in patient portal reviews [40].

### Conclusions

The TOPCOP taxonomy aims to support health information managers in comparing and selecting patient portals. By providing a standardized terminology to describe various aspects of patient portals independent of clinical setting or country, the taxonomy will also be useful for advancing research and evaluation of patient portals. Since the health information managers contributed to the taxonomy’s development, we were able to improve the taxonomy’s quality and usefulness based on the users’ needs.

The taxonomy consists of a manageable number of characteristics and dimensions and is therefore flexible for future changes. If needed, new dimensions can be added or removed according to future technological development. Further, due to its flexible form, the users can adjust the taxonomy to their personal needs. The initial TOPCOP taxonomy was developed by analyzing patient portals from 15 countries worldwide. It was then improved by health information managers with various degrees of patient portal experience from 3 countries with different levels of health care digitization. We, therefore, consider our taxonomy suitable to compare and classify patient portals from any country. The taxonomy may also contribute to the progress of health care digitization as it may enhance human resources capacity and effectiveness.

## Appendix

Multimedia Appendix 1 Achieved consensus on existing dimensions after round 1.

Multimedia Appendix 2 Consensus on new characteristics and dimensions after round 2 presented to the panelists in round 3.

Multimedia Appendix 3 Presentation of a proposal of a new characteristic to refine an existing dimension.

Multimedia Appendix 4 A detailed description of dimensions and characteristics.

## Abbreviations

EHR: electronic health record
PHR: personal health record
RAM: RAND/UCLA Appropriateness Method
TED: Taxonomy-Evaluation-Delphi approach.
TOPCOP: Useful Taxonomy of Patient Portals based on Characteristics of Patient Engagement
