# Supplementary material for: Improvement and Evaluation of the TOPCOP Taxonomy of Patient Portals: Taxonomy-Evaluation-Delphi (TED) Approach
Source: J Med Internet Res. 2021 Oct 5;23(10):e30701. doi: 10.2196/30701 (PMC8527386; doi:10.2196/30701)
Supplement: Multimedia Appendix 1 [file jmir_v23i10e30701_app1.pdf]

Multimedia Appendix 1. Achieved consensus on existing dimensions after round 1.

MEDIAN	DIMENSIONS	CHARACTERISTICS		
9	D1: Care Sector Target	primary care	secondary care	generic
8	D2: Portal Specialization	universal	disease-specific	
8	D3: Activity Monitoring	no insight	with insight	
8	D4: Patient Target	outpatient	in & outpatient	
9	D5: Appointment Booking	no booking	request	schedule
8	D6: Prescription Renewal	no renewal	basic renewal	advanced renewal
8	D7: Portal Customizability	not customizable	customizable	
9	D8: E-Consult	no e-consult	asynchronous	synchronous
8	D9: System Notifications	no notifications	reminder	alerts
9	D10: Patient Education	no education	non-personalized	personalized
8	D11: Therapy Instructions	non-protocol-based	protocol-based	
9	D12: Health Monitoring	no monitoring	self-reported	self-tracked
8	D13: Visit Preparation	no preparation	with preparation	
8	D14: Declaration of Will	no registration	with registration	
7	D15: Second Opinion	no inquiry	with inquiry	
7	D16: Study Sign-Up	no sign-up	with sign-up	
9	D17: Record Access	no control	shared control	full control
8	D18: Records Management	no management	with management	
9	D19: Health Data Amend	review	correct	delete
9	D20: Health Data Upload	no upload	with upload	
