# Supplementary material for: Improvement and Evaluation of the TOPCOP Taxonomy of Patient Portals: Taxonomy-Evaluation-Delphi (TED) Approach
Source: J Med Internet Res. 2021 Oct 5;23(10):e30701. doi: 10.2196/30701 (PMC8527386; doi:10.2196/30701)
Supplement: Multimedia Appendix 2 [file jmir_v23i10e30701_app2.pdf]

Multimedia Appendix 2. Consensus on new characteristics and dimensions after round 2 presented to the panelists in round 3

MEDIAN	DIMENSIONS	CHARACTERISTICS			
8	D1: Care Sector Target	primary care	secondary care	tertiary care	generic
7	D2: Portal Specialization	universal	extended	disease-specific	
	D3: Activity Monitoring	no insight	with insight		
	D4: Patient Target	outpatient	in & outpatient		
9	D5: Appointment Booking	no booking	request	schedule	hybrid
8	D6: Prescription Renewal	no renewal	with renewal		
	D7: Portal Customizability	not customizable	customizable		
8	D8: E-Consult	no e-consult	asynchronous	synchronous	both
7	D9: System Notifications	no notifications	notifications	reminder	alerts
	D10: Patient Education	no education	non-personalized	personalized	
8	D11: Therapy Instructions	no instructions	non-protocol-based	protocol-based	
9	D12: Health Monitoring	no monitoring	self-reported	self-tracked	combined
	D13: Visit Preparation	no preparation	with preparation		
	D14: Declaration of Will	no registration	with registration		
	D15: Second Opinion	no inquiry	with inquiry		
	D16: Study Sign-Up	no sign-up	with sign-up		
	D17: Record Access	no control	shared control	full control	
	D18: Records Management	no management	with management		
	D19: Health Data Amend	review	correct	delete	
	D20: Health Data Upload	no upload	with upload		
No consensus	D21: Account Protection	low	medium	strong	
7	D22: App Expandability	not expandable	expandable		
7	D23: Medical Specialty	generic	specialized		
8	D24: Medication Summary	no summary	with summary		
8	D25: Portal Type	tethered	integrated		
No consensus	D26: Web Accessibility	not supported	supported		

Legend
- New characteristic with consensus
- New dimension with consensus
- New characteristic without consensus
- New dimension without consensus
