# Supplementary material for: Improvement and Evaluation of the TOPCOP Taxonomy of Patient Portals: Taxonomy-Evaluation-Delphi (TED) Approach
Source: J Med Internet Res. 2021 Oct 5;23(10):e30701. doi: 10.2196/30701 (PMC8527386; doi:10.2196/30701)
Supplement: Multimedia Appendix 3 [file jmir_v23i10e30701_app3.pdf]

Multimedia Appendix 3. Presentation of a proposal of a new characteristic to refine an existing dimension.

DIMENSIONS	CHARACTERISTICS			
D1: Care Sector Target	primary care	secondary care	tertiary care	generic

Legend: [hatched box] proposed new characteristic. [empty box] existing characteristic.
