# Supplementary material for: Improvement and Evaluation of the TOPCOP Taxonomy of Patient Portals: Taxonomy-Evaluation-Delphi (TED) Approach
Source: J Med Internet Res. 2021 Oct 5;23(10):e30701. doi: 10.2196/30701 (PMC8527386; doi:10.2196/30701)
Supplement: Multimedia Appendix 4 [file jmir_v23i10e30701_app4.pdf]

Multimedia Appendix 4. Detailed description of dimensions and characteristics.

D1: Portal Type

A patient portal can have different degrees of integration. Health data can be gathered, aggregated, and displayed from only one healthcare provider, from healthcare organizations with multiple connected healthcare providers, or on a national level, where all healthcare providers transfer data from their health information system or EHR system to a national repository [28].

Tethered: A patient portal gathers and displays health data only from one healthcare provider.

Integrated: A patient portal gathers and displays health data from multiple healthcare providers.

D2: Care Sector

The dimension Care Sector refers to patient portals' different care sectors that may be specifically projected based on each care sector's individual needs.

Primary care: A patient portal is specifically projected for primary care providers (e.g., general practice offices). The patient portal may offer limited functionalities.

Secondary care: A patient portal is specifically projected for the secondary care provider (e.g., hospital inpatient care, acute care, specialists for advanced medical investigation and treatment) and may offer a broader range of functionalities suitable for the needs of this care sector.

Tertiary care: A patient portal is specifically projected for the tertiary care provider (e.g., rehabilitation facility, hospice, nursing home) and may offer a specific range of functionalities suitable for the needs of this care sector.

Generic: A patient portal is not designed for a specific care sector and offers more generic functionalities.

D3: Patient Target

The dimension Patient Target refers to the scope of a patient portal, which can be exclusively for outpatients or may offer additional features for inpatients and, in this case, support inpatients with specific features.

Outpatient: The patient portal is designed exclusively for outpatients and does not offer any features for inpatients.

In & outpatient: The patient portal is designed to be used by outpatients and inpatients. The patient portal offers additional features designed for inpatients. The inpatient features may be, for example, the creation of QR codes to check in to the hospital, floor layouts of the hospital, functionalities to order specific meals or manage room entertainment, or information on the health provider's staff and organization. Inpatient features are more likely to be included in tethered patient portals [135] [136] [137].

D4: Portal Specialization

The dimension Portal Specialization refers to a possible specialization of a patient portal (e.g., for specific diseases such as diabetes) and may provide specific features.

Universal: A patient portal can be developed for any kind of patient without any focus on, for example, diseases or patient groups.

Extended: The scope of a patient portal can be universal but may also offer some disease-specific features for one or several different diseases without having its scope on one specific disease.

Disease-specific: A patient portal is projected to fulfill patients' needs with specific diseases (e.g., diabetes or asthma). The scope is to provide specific features for one specific disease.

D5: Medical Specialty

A patient portal can provide specific features needed by healthcare providers offering medical specialty services such as allergy and immunology, neurology, orthopedics, pediatrics, or plastic surgery. While the dimension Portal Specialization refers to patient portals with the scope on one specific disease, patient portals can have a scope on medical specialty. By this, the portal offers specific features for related medical specialties.

Generic: The patient portal is designed without a specific scope on a medical specialty.

Specialized: The patient portal has a clear scope on one medical specialty and offers specific features for this particular medical specialty.

D6: Web Accessibility

A patient portal can be designed to offer web accessibility or not. Web accessibility ensures that there are no barriers that prevent interaction with or access to the patient portal by people with physical disabilities. Web accessibility may refer to the interface of the patient portal, to the health data, or to the method of communicating and interacting.

Not supported: The patient portal does not support web accessibility.

Supported: The patient portal supports web accessibility.

D7: App Expandability

A patient portal can offer a web application programming interface to connect third-party applications to enhance functionality and services. Adding third-party applications may be restricted to the healthcare provider or be permissible to the patient.

Not expandable: The patient portal does not offer a web application programming interface.

Expandable: The patient portal offers a web application programming interface.

D8: Activity Monitoring

The dimension Activity Monitoring refers to the functionality of allowing patients to monitor who accesses their health data.

No insight: The patients cannot monitor who accesses their health data.

With insight: The patients can monitor who accesses their health data (e.g., by accessing a log protocol, by receiving an automated dashboard message, or by receiving an automated email or text message). Depending on the portal set-up, the patients can monitor which stakeholder (e.g., physician, healthcare provider, or caregiver) has accessed their health data, which health data was accessed, and when it was accessed. Activity monitoring can be found in both patient portals where the patients can manage the health data access and where the patients cannot manage the health data access.

D9: Appointment Booking

This dimension refers to how patients can book an appointment through the patient portal with their health provider. Appointments can refer, for example, to a follow-up

doctor's visit or to a visit for a diagnostic procedure such as magnetic resonance imaging, radiography, ultrasound, or endoscopy.

No booking: The patient portal offers no functionality to book an appointment.

Request: The patient sends a message to the health provider through the patient portal to inquire about an appointment. The health provider's team checks availability and confirms the appointment with some delay. The same method applies to cancel an appointment.

Schedule: The patients can manage their appointments online in real-time. They select available dates from an online calendar, and the appointment is confirmed immediately by the system. The same method applies to cancel an appointment. Additional services can be an overview of all past, upcoming, missed, or canceled appointments.

Hybrid: The patient portal offers both methods of appointment booking depending on the type of appointment needed. Appointments for a regular doctor's visit may be scheduled in real-time, while appointments for diagnostic procedures may be done upon request.

D_{10}: Prescription Renewal (new)

This dimension refers to the functionality to renew a prescription through the portal.

No renewal: The patient portal offers no functionality to renew a prescription.

With renewal: The patient portal offers the functionality to request a prescription renewal through the patient portal.

D_{11}: Portal Customizability

The dimension Portal Customizability refers to the functionality that users can customize the language or content of the web interface composition according to their needs.

Not customizable: The patient cannot adjust the patient portal's interface or language. Simple changes of the web interface, such as change of font size or colors, refer to this characteristic.

Customizable: The patient portal has functionalities to customize the web interface. Patient portals present the information in a predefined way (e.g., the collected vital signs of the patient). These views may contain too much or too little information and the presentation may not be satisfactory to the patient. Therefore, some patient

portals offer the possibility for patients to adjust which information is displayed and how. Patients can, for example, change the granularity of the information, include additional information or exclude information, adjust the order of the information, modify the information's language, or alter chart types.

D12: E-Consult

The dimension E-Consult refers to the functionality that a patient can receive medical consultation through the patient portal.

No e-consult: The patient portal offers no functionality to receive medical consultation through the patient portal.

Asynchronous: The patients can communicate with their health provider through secure messaging to receive medical consultation. Asynchronous communication implies a time delay in answering the patient's questions and is therefore an asynchronous consultation.

Synchronous: The patient can receive medical consultation (e.g., via video conferencing or online chat). Since there is no time delay in communication, the methods are characterized as synchronous consultation.

Both: The patient can opt for an asynchronous or synchronous medical consultation as described above.

D13: System Notifications

The dimension System Notifications refers to the sending of automated messages to the patients based on events. Notifications, reminders, and alerts have different scopes and different technical and organizational requirements.

No notifications: The patient portal system does not send any automated messages to the patient (except notifications related to the registration process).

Notifications: The aim is to inform the patient related to an event. Informing can refer, for example, to notifying patients about new documents or information added to their electronic health record, a change of the treating physician, stakeholders having accessed their EHR, documents needed from the patient for the upcoming visit, or information the patient needs to read before an upcoming visit.

Reminder: The focus is to remind the patient. Notifications are sent to remind patients, for example, of an upcoming appointment with their physician or health

maintenance, such as medicine intake, immunization, or mammography screening. The functionality of reminders also covers notifications.

Alerts: The focus is to alert a patient in case of anomalies or hazards. Events for alerts can include warnings about possible adverse drug events or a changing health status recognized by wearables or smartphone sensors connected to the patient portal system. Patient portals that offer alerts commonly also provide the functionality of sending notifications and reminders.

D_{14}: Patient Education

The dimension Patient Education refers to educational material provided to the patient through the patient portal.

No education: The patient portal does not provide any educational material.

Non-personalized: The patient portal provides access to a health library, for example, but does not provide processed information related to the individual health situation of the patient.

Personalized: The patient portal provides protocol-based health information related to the individual health situation of the patient. By this, patients with diabetes, for example, receive health education material and personal decision support according to their specific health problem. The protocol-based processing and providing of the information is done by the provider's health information system, while the patient portal is set up to display the information.

D_{15}: Therapy Instructions

Patient portals can have the functionality to send protocol-based therapy and lifestyle instructions to the patient.

Non-instructions: The patient portal does not offer specific functionality to provide therapy and lifestyle instructions to the patient.

Non-protocol-based: The patient portal does not automatically generate individual therapy and lifestyle instructions. The physician creates the therapy and lifestyle instructions manually that can be viewed by the patient inside a specific section.

Protocol-based: The provider's health information system generates protocol-based individual therapy and lifestyle instructions based on health data entered by the physician or the patient (e.g., self-reported or self-tracked health data). The triggered instructions are related to the patient's health profile and the data entered. The

patient portal is technically and design-wise set up to transmit personal generated health data to the provider's health information system and display the therapy and lifestyle instructions.

D16: Medication Summary

The patient portal can offer the functionality of an online medication summary collecting the prescriptions of one or multiple healthcare providers. The patient portal can offer supplementary functions such as

No summary: The patient portal offers no medication summery.

With summary: The patient portal provides a medication summary. Additional functionalities of a medication summary may be, for example, personalized intake recommendation, online monitoring of drug interactions, online forms where the patient can communicate the actual intake, online forms to report perceived side effects.

D17: Health Monitoring

The dimension Health Monitoring relates to the portal's functionality to upload patient-monitored health data to the patient portal.

No monitoring: The patient portal does not offer any functionality to report self-monitored or self-reported health-related data.

Self-reported: The patient portal offers specific e-journals or a patient diary to report patient-generated health data (e.g., blood pressure measures, blood glucose value, cholesterol value, or information on well-being, pain, or mental and physical condition). The patient uploads the collected vital data or lifestyle habits manually to specific e-journals or to a patient diary.

Self-tracked: The patient portal provides the technical requirements to connect wearables and smartphone sensors that track and monitor the patient's vital signs. These gadgets connect via the internet with the patient portal system and automatically transmit the collected data, which physicians can then access. Self-tracked health data may also be added to a patient diary.

Combined: The patient portal provides both options (self-reported and self-tracked) to report personal generated health data.

D18: Visit Preparation

The dimension Visit Preparation refers to the functionality to provide the physician in advance with requested information through the patient portal. A patient portal may offer a structured sequence of e-journals to be filled out to prepare the visit with the information requested by the physician. These e-journals can be universal or related to the treatment and aim to provide initial or extra information on the patient's health status. The e-journals may vary in terms of the degree of structured recording options and may allow the uploading of documents. The patient portal may also provide e-journals to give patients the option of submitting questions to be discussed during the visit or requests for specific treatments. Data shared in this way usually does not become part of the EHR. Further, hospitals can offer specific e-journals to collect all needed information for the patient's intake.

No preparation: There are no specific functionalities offered to prepare the visit.

With preparation: There are specific functionalities to prepare the visit with the physician, the patient's hospital intake, or e-journals to submitting questions to be discussed during the visit.

D_{19}: Declaration of Will

This dimension refers to the functionality that a patient can communicate advanced directives (e.g., to accept or decline blood transfusions, draft a living will, authorize organ donations, or donate their body). It also includes permitting the use of the patient's health data for research.

No registration: The patient portal does not offer a specific functionality that the patient can enlist for advanced directives.

With registration: The patient portal offers the specific functionalities that the patient can enlist for advanced directives. This may include a secure methodology for the verification of the patients' identity.

D_{20}: Second Opinion

This dimension refers to the possibility that patients can select another physician through the patient portal and share their health data to inquire a second opinion.

No inquiry: The patient portal does not offer the functionality to inquire a second opinion online.

With inquiry: The patient portal offers the functionality to inquire a second opinion online.

D21: Study Sign-Up

This dimension refers to patients receiving information on studies matching their disease and the functionality to participate in matching studies. Patients can grant permission to use their health data for research.

No sign-up: The portal does not offer any functionalities to participate in studies.

With sign-up: The portal offers functionalities to participate in studies by signing up.

D22: Record Access

The dimension Record Access refers to the extent to which patients can grant access to their health data to a health provider and other stakeholders (e.g., caregivers or family members). This dimension refers to who can access which health data of a patient.

No control: The access to the health data for all stakeholders is controlled exclusively by the health provider and not by the patient. This functionality can be commonly found in tethered patient portals.

Shared control: Patients can grant access to their health data for some but not all stakeholders. Depending on the patient portal settings, the patient can grant access to caregivers, for example, while the health provider controls physicians' or health providers' access.

Full control: Patients can decide who can access their health data. The patients can grant or block access for all or selected stakeholders (e.g., health provider, physicians, or caregiver) or hide individual documents from one or all stakeholders. This functionality can be found in integrated patient portals of health organizations or national patient portals. This functionality moves the strict EHR concept where the provider manages the access to the health data to a more patient-centric health record.

D23: Records Management

This dimension refers to the functionality that patients can manage whether a health provider can add data to the patient portal's EHR or not. This functionality may also include setting privilege rights as to what kind of health data can be added.

No management: Patients cannot manage who can add health data to the patient portal's EHR.

With management: Patients can manage who can add health data to the patient portal's EHR. This functionality moves the strict EHR concept where the management health data belongs to the provider to a more patient-centric health record and can be found in integrated patient portals of health organizations and national patient portals [138]. Note: The dimension Record Access refers to granting access rights to read a patient's health data, while Records Management refers to granting access rights to add health data and modify the data.

D24: Amend Health Data

The dimension Amend Health Data refers to the functionality that a patient can correct or delete health data held in the patient portal. Changes refer to the data held in the patient portal's repository, and changes usually do not affect the EHR of a health provider.

Review: Patients can only review the data added by the health provider but cannot make any changes on their own. In this case, some portals offer the functionality of an amend request to communicate the wrong information and request an update of the health data from the health provider [139].

Correct: Patients can modify selected health data on their own. The functionality to correct usually refers to information on allergies, immunization, or anamnesis but may also refer to other health data.

Delete: Patients can delete single documents held in the patient portal's repository. The deletion affects only the patient portal's repository and not the data held in the health provider's EHR system. This functionality can be found, for example, in integrated patient portals of health organizations or national patient portals [28].

D25: Health Data Upload

This dimension refers to the functionality that a patient can upload health data created by other health providers (e.g., medical images, care summaries, referral letters, treatment plans), which become part of the EHR. This functionality is different from the functionality of the dimension Visit Preparation as that data does not integrate into the EHR.

No upload: The patient portal does not offer the functionality to upload health data into the EHR.

With upload: The patient portal offers the patient the possibility to upload health data into the EHR. This functionality moves the strict EHR concept to a more patient-centric patient portal that operates under collaborative models, combining content from individuals and healthcare professionals depending on the health record's purpose.
